# The best fixation target revisited: New insights from retinal eye tracking

**DOI:** 10.3758/s13428-025-02890-1

**Published:** 2025-11-19

**Authors:** Diederick C. Niehorster, Szymon Tamborski, Marcus Nyström, Robert Konklewski, Valentyna Pryhodiuk, Krzysztof Tołpa, Roy S. Hessels, Maciej Szkulmowski, Ignace T. C. Hooge

**Affiliations:** 1 Lund University Humanities Lab, Lund University, Lund, Sweden; 2 Department of Psychology, Lund University, Lund, Sweden; 3 Department of Biophotonics and Optical Engineering, Institute of Physics, Faculty of Physics, Astronomy and Informatics, Nicolaus Copernicus University, Toruń, Poland; 4 Inoko Vision, Toruń, Poland; 5 Experimental Psychology, Helmholtz Institute, Utrecht University, Utrecht, The Netherlands

**Keywords:** Eye tracking, Fixation, Retinal, High precision, Saccade, Drift, Fixational eye movement, Stability

## Abstract

In many tasks, participants are instructed to fixate a target. While maintaining fixation, the eyes nonetheless make small fixational eye movements, such as microsaccades and drift. Previous work has examined the effect of fixation point design on fixation stability and the amount and spatial extent of fixational eye movements. However, much of this work used video-based eye trackers, which have insufficient resolution and suffer from artefacts that make them unsuitable for this topic of study. Here, we therefore use a retinal eye tracker, which offers superior resolution and does not suffer from the same artifacts to reexamine what fixation point design minimizes fixational eye movements. Participants were shown five fixation targets in two target polarity conditions, while the overall spatial spread of their gaze position during fixation, as well as their microsaccades and fixational drift, were examined. We found that gaze was more stable for white-on-black than black-on-grey fixation targets. Gaze was also more stable (lower spatial spread, microsaccade, and drift displacement) for fixation targets with a small central feature but these targets also yielded higher microsaccade rates than larger fixation targets without such a small central feature. In conclusion, there is not a single best fixation target that minimizes all aspects of fixational eye movements. Instead, if one wishes to optimize for minimal spatial spread of the gaze position, microsaccade or drift displacements, we recommend using a target with a small central feature. If one instead wishes to optimize for the lowest microsaccade rate, we recommend using a larger target without a small central feature.

## Introduction

When attempting to fixate a target as steadily as possible, the eyes still exhibit slow continuous motion called ocular drift, which is interspersed with small saccades, often called microsaccades (Rolfs, 2009; Poletti, 2023). Studies of these fixational eye movements have found that they may perform an important role in perception (e.g., Hafed, 2013; Boi, Poletti, Victor, & Rucci, 2017; Clark, Intoy, Rucci, & Poletti, 2022; Rolfs & Schweitzer, 2022; Poletti, 2023; Nghiem, Witten, Dufour, Harmening, & da Silveira, 2025), may reflect the allocation of attention (Hafed & Clark, 2002; Engbert & Kliegl, 2003; Hafed, Lovejoy, & Krauzlis, 2011; Khademi et al., 2024) and may also be relevant clinically (e.g., Kumar & Chung, 2014; Chung, Kumar, Li, & Levi, 2015; Shaikh, Otero-Millan, Kumar, & Ghasia, 2016; Sheehy et al., 2020; Nieboer, Ghiani, de Vries, Brenner, & Mann, 2023; Murari et al., 2024; Lee et al., 2025). In many situations, these fixational eye movements are, however, a nuisance. For instance, during ophthalmologic imaging, the continuous movements of the eye reduce image quality (Carrasco-Zevallos et al., 2016; de Castro et al., 2023), during eye surgery, they negatively impact outcomes (Mrochen, Kaemmerer, Mierdel, & Seiler, 2001; Porter et al., 2005), and they introduce noise in studies of retinal function (Sutter & Tran, 1992; Menz, Sutter, & Menz, 2004; Zhang et al., 2008). In these and many other settings, it may thus be desired to minimize fixational eye movements.

Fixation targets are often used to elicit stable fixation, i.e., fixation during which there is minimal fixational eye movement. Many different fixation target designs have been described in the literature (see Thaler, Schütz, Goodale, & Gegenfurtner, 2013, for a systematic overview; and see also Nieboer et al., 2023), but it remains unclear which fixation target design elicits the most stable fixation. A number of studies have investigated the impact of various design parameters of fixation targets on fixation stability. Most of these studies investigated overall fixation stability, operationalized as the dispersion of the gaze position (quantified as the BCEA, Crossland & Rubin, 2002, or as standard deviation of gaze positions) during the entire fixation episode. Some also looked at the rate of microsaccades or the displacement of the microsaccades that occurred during the fixation episode. Several studies reported that target contrast, target color, background color, and background shapes did not affect the overall fixation stability (Steinman, 1965; Boyce, 1967; Murphy et al., 1974; Ukwade & Bedell, 1993). Fixation was less stable only if target contrast was such that the fixation target was barely visible or not visible at all (McCamy, Najafian Jazi, Otero-Millan, Macknik, & Martinez-Conde, 2013; see also; Cherici, Kuang, Poletti, & Rucci, 2012). The size of the fixation target has a modest impact on overall fixation stability, with smaller fixation targets yielding less gaze dispersion during fixation and smaller microsaccades (Steinman, 1965; Rattle, 1969; McCamy et al., 2013; Thaler et al., 2013; Hirasawa et al., 2016). There are, however, inconsistent reports of the effect of target size on microsaccade rate, with one study reporting a lower microsaccade rate for smaller targets (Thaler et al., 2013), while other studies reported higher microsaccade rates for smaller targets (McCamy et al., 2013; Steinman, 1965). Target blur leads to lower overall fixation stability and more microsaccades (Ghasia & Shaikh, 2015; Ukwade & Bedell, 1993). Fixation of more complex targets consisting of multiple features (a circle with a cross and a small point, Thaler et al., 2013; and a Bessel function consisting of a central lobe surrounded by multiple rings of decreasing intensity, Bhattarai, Suheimat, Lambert, & Atchison, 2019) has been reported to be more stable than fixation of simple shapes (a circle, Thaler et al., 2013 and a Gaussian blob, Bhattarai et al., 2019). Finally, Bowers et al. (2021) reported that an animated fixation target consisting of inward shrinking concentric circles did not yield different overall fixational stability, saccade, or drift characteristics than two static target designs (a Maltese cross and a disk), nor did the static targets yield differences in fixational eye movements.

Another noteworthy difference between the above studies is in the eye-tracking technology used to study fixation stability. While many of the older studies used various techniques that offer very high precision, much of the more modern work used video-based eye trackers. We question whether video-based eye trackers are suitable for studying the stability of fixation because they suffer from several important drawbacks. First, video-based eye trackers are not suitable to study ocular drift because even though they may produce gaze signals that resemble ocular drift, these signals likely for a significant part do not reflect physical eye movements, but instead artefacts of the eye tracking method (see Niehorster, Zemblys, & Holmqvist, 2021, for a discussion). Specifically, small movements of the head with respect to the eye tracker camera may induce changes in recorded gaze position (Merchant et al., 1974; Houben et al., 2006; Kolakowski & Pelz, 2006; Cerrolaza et al., 2012; Hermens, 2015; Holmqvist et al., 2021), while changes in pupil size may also yield shifts in the gaze position reported by the eye tracker in the absence of actual eye rotation (pupil-size artefact, PSA, see Wyatt, 2010; Drewes, Masson, & Montagnini, 2012; Drewes, Zhu, Hu, & Hu, 2014; Choe, Blake, & Lee, 2016; Jaschinski, 2016; Hooge, Hessels, & Nyström, 2019; Hooge, Niehorster, Hessels, Cleveland, & Nyström, 2021; Hooge et al., 2025). Second, video-based eye trackers do not have the resolution required to capture the smallest microsaccades and lack sufficient precision (RMS-S2S of about 2.4$^{\prime }$ or worse, Nyström, Niehorster, Andersson, & Hooge, 2021) to accurately assess slow fixational eye movements such as ocular drift (Collewijn & Kowler, 2008; Kimmel et al., 2012; McCamy et al., 2015; Poletti & Rucci, 2016). One exception is Bowers et al. (2021), who used a retinal eye tracker, but focused their study on the effect of fixation task (active, where participants performed a discrimination task at the fixation location vs passive, participants were instructed to fixate without a further task) on fixation stability, not on the effect of fixation point design.

Given these concerns (the presence of artefactual drift due to PSA and head movement, and insufficient resolution and precision), we think that a significant part of the work cited above is in need of re-examination. Specifically, we cannot rule out that part of the reported differences in fixational stability are artefacts of the employed eye tracking method (P-CR video-based eye tracking), while potentially interesting effects of fixation target design on fixational stability may have been missed. Here, we therefore, like Bowers et al. (2021), use a retinal eye tracker to reexamine the stability of fixation enabled by a range of fixation targets used in previous studies (Thaler et al., 2013; Bhattarai et al., 2019). The gaze position reported by the retinal eye tracker we used (Bartuzel et al., 2020) is not affected by small head movements or changes in pupil size (Hooge et al., 2025). It furthermore offers a precision that is far superior to that of video-based eye trackers (RMS-S2S as low as 0.07$^{\prime }$, see methods below, compared to around 2.4$^{\prime }$ for even the best video-based eye trackers, Nyström et al., 2021), enabling us to not only examine overall fixation stability using the bivariate contour ellipse area (BCEA, a common measure of spatial spread of gaze positions during fixation, Steinman, 1965; Crossland & Rubin, 2002; Castet & Crossland, 2012; Niehorster, Zemblys, Beelders, & Holmqvist, 2020), but also to separately investigate the properties of the saccades and ocular drift that occur during fixation.

The current study had two research questions. First, we asked whether we could replicate the results of previous studies (Thaler et al., 2013; Bhattarai et al., 2019) regarding which is the ‘best’ fixation target, i.e., the target that yields the most stable fixation in terms of lowest spread of gaze positions during fixation and lowest microsaccade rate. Capitalizing on the sensitivity of our measurement setup, we furthermore extend the examination of what is the ‘best’ fixation target by systematically comparing the characteristics of microsaccades and ocular drift between the different fixation targets. Given the above-reviewed literature, we expect that fixation targets that include a small feature (such as a dot at their center), and those that contain multiple features (such as multiple rings or an additional cross) will elicit more stable fixation in the sense of a lower spread in recorded gaze positions. Whether such targets will also yield a lower microsaccade rate is unclear since previous studies (Steinman, 1965; McCamy et al., 2013; Thaler et al., 2013) have reported different effects of target size on microsaccade rate. Our second research question was whether fixation stability is affected by the polarity of the fixation target with respect to the background on which it is placed. Specifically, we both placed white targets on a black background, and black targets on a grey background – two types of fixation displays that are commonly used in the literature. We did not expect systematic differences between these two polarity conditions, since previous studies reported that target contrast, polarity, and background luminance did not affect fixational stability (Steinman, 1965; Boyce, 1967; Ukwade & Bedell, 1993; McCamy et al., 2013). It may, however, be possible that fixation would be more stable for targets on a grey background because, in our setup, the outer edge of the grey background had high contrast with the black edge of the screen, whereas for the black background condition, this edge was not visible. This edge in the grey background condition provides an additional feature that may help stabilize gaze.

**Fig. 1:**
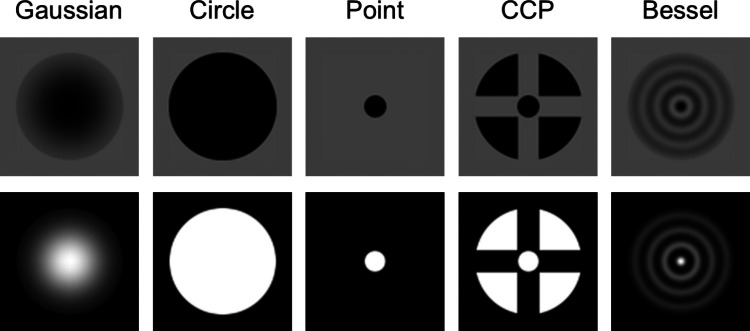
The five fixation target designs used in the study, each rendered in the black-on-grey (*top row*) and white-on-black (*bottom row*) target polarity conditions. CCP refers to the circle-cross-point target

## Method

### Participants

Six participants (26–59 years old, five males, one female) with normal or corrected to normal vision took part in the experiment and provided informed consent. Five are authors of the current article. Three were naive with respect to the previous research on fixation point design at the time of recording, but were experienced in viewing targets in ophthalmological devices. During the set up of the equipment, a Badal optometer system integrated in the eye-tracking setup (Bartuzel et al., 2020) was used to ensure that the visual stimuli appeared sharply focused to the participants.

### Apparatus and visual stimuli

Eye-tracking data were acquired using the FreezEye Tracker (FET, Bartuzel et al., 2020). This prototype device operates based on the confocal scanning laser ophthalmoscope principle and records small rectangular retinal frames measuring $(3.00^\circ \pm 0.05^\circ ) \times (3.00^\circ \pm 0.05^\circ )$ at a sampling rate of 620 Hz. Comparing the setup reported in Bartuzel et al. (2020) with the device used in our study, several significant improvements were made: (i) the light source of the tracker was changed from a laser diode (wavelength: 785 nm, optical power: 100 $\upmu$W) to a superluminescent diode (central wavelength: 920 nm, spectral width: 20 nm, optical power: 200 $\upmu$W) to make the scanning beam invisible for the participant, thereby avoiding potential unwanted distractions and decreasing the speckle contrast in the output images; and (ii) the back-illuminated paper screen for displaying a target was replaced with a video projector (Dell M410HD) which, coupled with a customized optical subsystem, featured an optical resolution of 0.9$^{\prime }$ (approximately 67 pix/deg, which means individual pixels were not visible) and enabled presenting a series of images to implement this study’s protocol. Gaze trajectories are computed through pairwise alignment of sequential retinal imaging frames acquired by the FET device. The FET demonstrates high precision, the root mean square sample-to-sample deviation (Niehorster et al., 2020, RMS-S2S) of the data used in this article was 0.156$^{\prime }$ (range 0.07$^{\prime }$–0.60$^{\prime }$). Notably, the FET does not require calibration, except for the zero point. Average data loss (retinal frames for which a gaze position could not be determined), including blinks, was 0.65% (range 0–7.1%) for the data used in this article.

Visual stimuli were presented using a Dell M410HD projector configured in vertically inverted mode, as determined by the optical channel characteristics. The effective display resolution was 800 pixels $\times$ 1280 pixels, with a refresh rate of 59.81 Hz. A subset of the screen measuring 400 pixels $\times$ 400 pixels (corresponding to $6^\circ \times 6^\circ$ of visual angle) was used for stimulus presentation. To minimize head movements, participants were stabilized using a chin and forehead rest. Stimuli were presented using PsychoPy version v2023.1.0 (Peirce, 2007; Peirce et al., 2019).

Five different fixation target designs were used, differing in sharpness of their edges, size of their features and the number of features (Fig. 1). Specifically, from Bhattarai et al. (2019) the Gaussian circle and Bessel fixation targets were used, and from Thaler et al. (2013) the Circle (their B), Point (their A) and Circle-Cross-Point (their ABC, hereafter called CCP) were used. The outer diameter of all fixation targets except the Point target was $1.02^\circ$. The diameter of the Point target, as well as the point at the center of the CCP target was $0.21^\circ$. Each was rendered in two target polarity conditions, black-on-grey, and white-on-black. The Bessel target was generated using a Bessel function of the first kind, implemented using the besselj() function in MATLAB R2023b.

### Procedure

At the beginning of each recording, participants were seated and placed their head on the chin- and forehead rest in front of the eyepiece through which the displays were viewed. They then adjusted the position of the chinrest, and the focus of the Badal optometer system until they had a clear image of the stimulus. Then, the operator adjusted the focus of the FET imaging path to optimize the retinal image, and recording commenced. Each recording session started with a series of 2-s presentations of a small CCP target (outer diameter $0.3^\circ$, center point diameter $0.06^\circ$) placed at $2.82^\circ$ from the center of the screen in the $45^\circ$, $135^\circ$, $225^\circ$, and $315^\circ$ directions. After each of these eccentric targets, the same small CCP target was displayed at the center of the screen for 2 s. Gaze data during the four fixations on the small CPP target at the center of the screen were used to calibrate the zero-point of each recording. After this sequence of eight 2-s fixation screens, the five fixation targets were shown in random order for 30 s each. Between each fixation target, masks consisting of randomly placed segments of the five fixation targets were shown for 3 s.

Each participant completed at least three sessions per polarity condition, with the fixation targets presented in different random orders. For some participants, more sessions were conducted to have a buffer in case some of the recordings with the prototype eye tracker failed. All available successful sessions (up to six) were used in the analysis.

### Data analysis

First, the recorded eye-tracking data for a session was split into five 30-s segments, one per fixation target. Microsaccades in each of these segments were independently detected using the Engbert and Kliegl algorithm (Engbert & Kliegl, 2003) using default parameters and a $\lambda$ of 6 and a minimum saccade displacement of 4$^{\prime }$. Saccades closer together than 20 ms were merged to avoid oscillations at the end of saccades being detected as separate saccades. All episodes between microsaccades that did not contain data loss and were longer than 100 ms were coded as drift episodes. Saccades and drift episodes during the first half second of a segment were discarded from further analysis to avoid including in the analysis potential saccades executed to acquire the target after the mask interval between fixation target displays. Furthermore, saccades starting or landing more than $2.5^\circ$ from the center of the fixation target, as well as drift episodes with a mean position more than $2.5^\circ$ from the center of the fixation target were discarded from further analysis. Across all measurement sessions, a total of 19 saccades and four drift episodes were excluded from further analysis using this distance criterion, which corresponds to 0.4% of all saccades and 0.1% of all drifts included in the analysis.

**Fig. 2:**
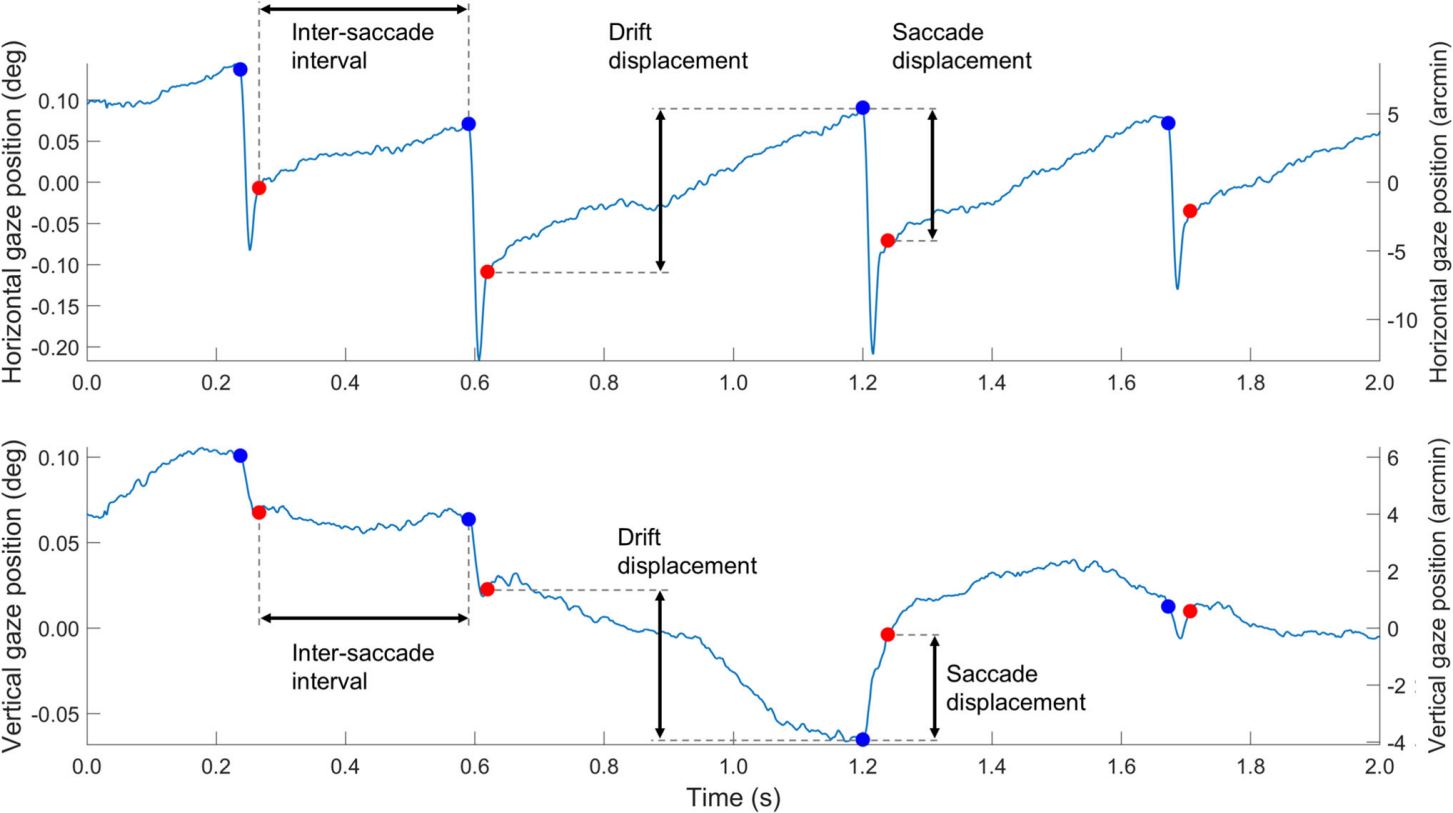
Example gaze position data over time. Indicated are the starts (*blue dots*) and ends (*red dots*) of detected microsaccades, and graphical representations of the operationalization of microsaccade and drift displacement. Also indicated is the inter-saccade interval, which is roughly reciprocal to the microsaccade rate

The selected microsaccades and drift episodes were further analyzed. First, the Bivariate Contour Ellipse Area (BCEA, Steinman, 1965; Crossland & Rubin, 2002; Castet & Crossland, 2012; Niehorster et al., 2020) for the gaze positions during the selected microsaccade and drift episodes was computed. The BCEA ellipse was scaled such that it contained 68.27% of the gaze positions (i.e., 1 standard deviation). The BCEA lumps together the microsaccade and drift components of fixational eye movements. To examine in more detail which aspects of fixational eye movements differ for the different fixation targets, we computed several metrics for the microsaccades and drift episodes (see Fig. 2 for operationalizations of several of these metrics).

For the microsaccades, the rate, displacement, and direction were computed. Microsaccade displacement was operationalized as the distance between the last gaze position sample before the start of the microsaccade and the first gaze position sample after the end of the microsaccade. As such, it did not include the overshoot at the end of the microsaccade. The direction of each microsaccade was determined using the same gaze position samples as microsaccade displacement.

For the drift episodes, the displacement, direction, path length, and speed were determined. Drift displacement was operationalized as the distance between the first gaze position sample at the start of the drift episode and the last gaze position sample at the end of the drift episode. The direction of drift during each episode was determined using the same gaze position samples as drift displacement. Path length was determined by computing the distances between adjacent samples during a drift episode and then summing these distances. To determine drift speed, first the speed along each axis (horizontal and vertical) was computed separately by calculating the gradient of the position signal using the gradient() function in the Python package numpy, employing second-order central differences. Drift speed was then operationalized as the mean magnitude of the gaze velocity signal during a drift episode. The duration of drift episodes was not examined as it is reciprocal to the microsaccade rate, and was thus redundant.

To visualize the distribution of microsaccade and drift displacement and direction, computed displacements and directions were collected per fixation target, polarity condition and participant and submitted to a 2D kernel density estimation procedure (Botev et al., 2010). The output was then discretized into a 22-level contour plot of which the lowest level was not shown for clarity.

**Fig. 3:**
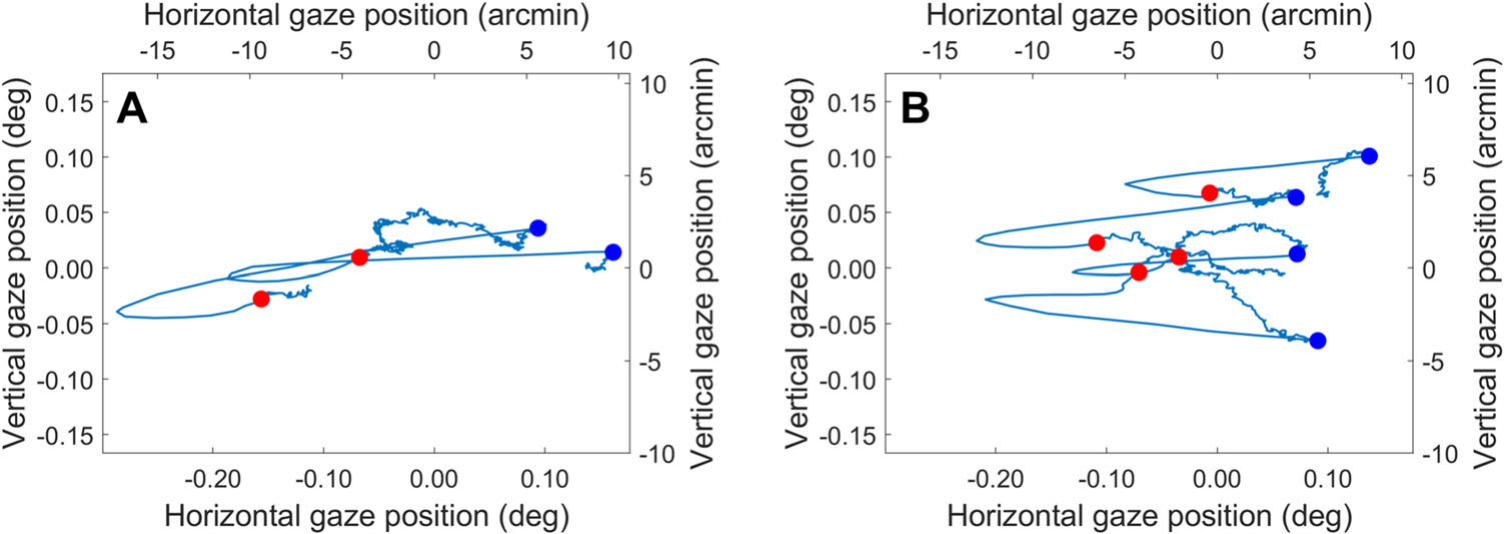
Example gaze position data. Each panel contains 2 s of representative data from participant P1 recorded while they viewed *A* the Gaussian fixation target and *B* the Bessel fixation target. Indicated are the starts (*blue dots*) and ends (*red dots*) of detected microsaccades. The data in panel B are the same data as is shown as a timeseries in Fig. 2

To examine whether the fixation targets yielded differences on any of the examined fixational eye movement measures, we performed Bayesian ANOVA (Rouder et al., 2012; Morey & Rouder, 2015) analysis using JASP 0.19.3 (JASP Team, 2025). Specifically, 2 (polarity) x 5 (fixation target) repeated measures ANOVAs were conducted with participant as a random factor. We adopt the notation for Bayes factors as implemented in JASP, which quantify the strength of evidence in favor of a difference between conditions given the observed data. Higher Bayes factor values indicate stronger evidence (Schönbrodt & Wagenmakers, 2018); Bayes factors in the range of 3–10 are interpreted as providing moderate evidence, values between 10 and 30 as strong evidence, values between 30 and 100 as very strong evidence, and those exceeding 100 are considered to indicate extreme evidence in favor of the tested hypothesis. Conversely, Bayes factors below 1 provide evidence against the tested hypothesis and thus indicate the strength of evidence that there is no difference between conditions. Specifically, Bayes factors between 0.1 and $\frac{1}{3}$ indicate moderate evidence against the hypothesis, between $\frac{1}{3}$ and 0.1 strong evidence, between 0.01 and $\frac{1}{30}$ very strong evidence, and values smaller than 0.01 extreme evidence against the tested hypothesis. Bayes factors within the range of $\frac{1}{3}$ to 3 indicate that the data do not provide substantial evidence either in favor of or against the hypothesis.

## Results

Figure 3 depicts 2 s of example data recorded for the Gaussian (Fig. 3A) and Bessel (Fig. 3B) fixation targets, which allows getting a sense for the data. As can be seen, it appears that the spatial spread of the whole gaze segment is less for the Bessel than for the Gaussian target, while microsaccades were both less frequent and larger for the Gaussian than for the Bessel target. These observations will be examined quantitatively based on the whole dataset below, along with examinations of further properties of fixational eye movements.

### Overall fixation stability

**Fig. 4:**
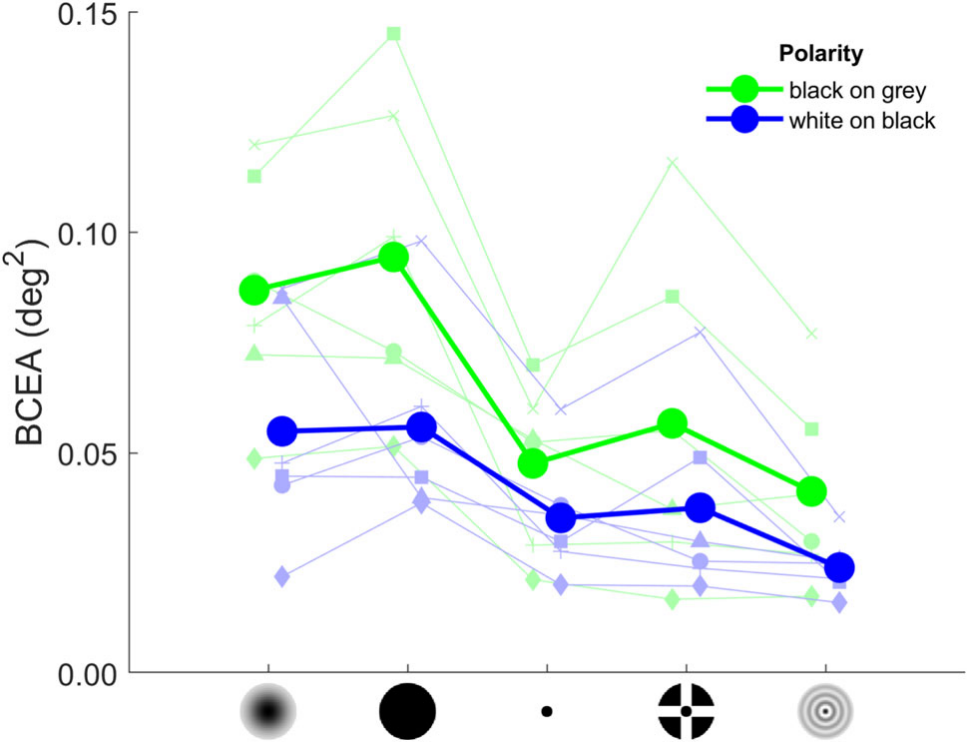
BCEA for the different fixation targets and polarity conditions. Shown are the BCEA for each fixation target and polarity conditions, averaged across participants (*large filled circles connected by thick lines*). Also shown are the values for the six individual participants (*small faded symbols connected by thin lines*). The data for the two polarity conditions are horizontally offset for clarity

We first examine overall fixation stability as assessed by the BCEA. Figure 4 shows the average BCEA for the different fixation targets and polarity conditions, along with the values for each participant. A Bayesian ANOVA analysis showed that a model with only the main effects of fixation target and target polarity was most supported by the data ($BF_m=4.17$). Overall, fixation stability was worse for black-on-grey targets (0.0654 deg$^2$) than for white-on-black targets (0.0415 deg$^2$, $BF_{10}>10,000$). Furthermore, pairwise comparisons showed that across both target polarities, fixation stability was worse for the Gaussian (0.0709 deg$^2$) and Circle (0.0752 deg$^2$) targets than for the other three fixation targets (Point: 0.0414 deg$^2$, CCP: 0.0471 deg$^2$, Bessel: 0.0326 deg$^2$; $BF_{10}>8.88$). Fixation stability was the same for the Gaussian and Circle targets ($BF_{10}=0.268$) and for the Point and CCP targets ($BF_{10}=0.269$). The data did not allow us to make a statement regarding whether there is a difference in BCEA between the Bessel target and the Point ($BF_{10}=0.928$) or CCP ($BF_{10}=1.71$) targets.

**Fig. 5:**
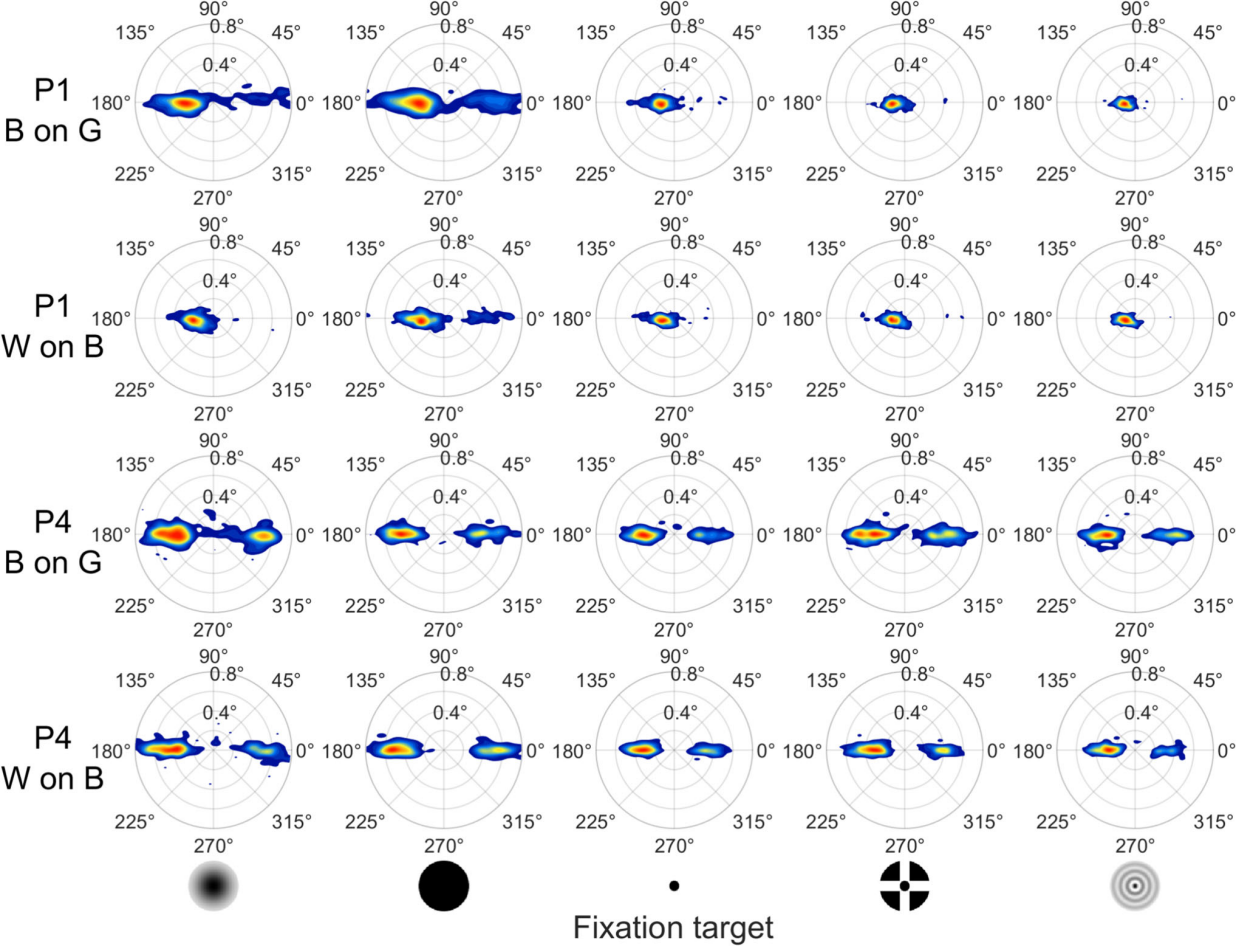
Histograms of microsaccade displacement and direction. Histograms showing microsaccade displacement and direction are plotted per fixation target and polarity condition for two example participants (P1 and P4). *B on G* refers to the black-on-grey target polarity condition, and *W on B* to the white-on-black target polarity condition

### Microsaccade characteristics

Next, we examine the characteristics of the microsaccades. Figure 5 provides example histograms of two representative participants showing the distribution of displacement and direction of microsaccades. As can be seen, saccades were mostly horizontal, with one participant making microsaccades predominantly in one direction (P1, leftward), while the other example participant made saccades in two directions (P4, leftward and rightward). Indeed, all participants showed saccades in either one (two participants) or two opposing (four participants) directions. Microsaccade displacement seems to be smaller for the targets containing smaller features (Point, CCP, and Bessel) than for the targets that do not contain such features (Gaussian and Circle), but does not appear to differ systematically between target polarities. Microsaccade direction does not appear to differ systematically between targets and target polarities.

**Fig. 6:**
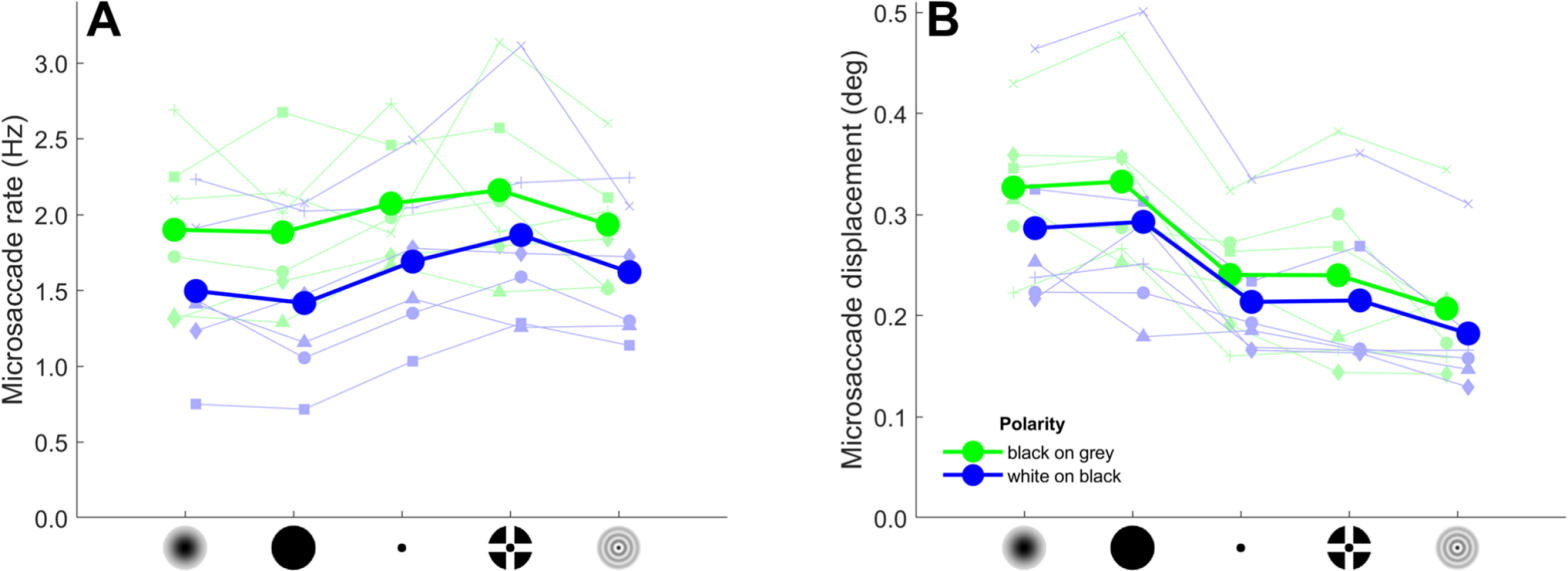
Microsaccade characteristics for the different fixation targets and polarity conditions. Shown are the microsaccade rate (**A**) and microsaccade displacement (**B**) for each fixation target and polarity condition, averaged across participants (*large filled circles connected by thick lines*). Also shown are the values for the six individual participants (*small faded symbols connected by thin lines*). The data for the two polarity conditions are horizontally offset for clarity

Figure 6 shows quantitative analyses of the microsaccade characteristics. A Bayesian ANOVA analysis of microsaccade rate (Fig. 6A) showed that a model with only the main effects of fixation target and target polarity was most supported by the data ($BF_m=12.2$). Overall, microsaccade rate was higher for black-on-grey (1.99 Hz) than for white-on-black targets (1.62 Hz, $BF_{10}>10,000$). Furthermore, pairwise comparisons showed that, across both target polarities, no large differences in microsaccade rate between targets are seen. Microsaccade rate was higher for the CCP target (2.01 Hz) than for the Circle target (1.65 Hz, $BF_{10}=3.32$). The microsaccade rate was the same for the Gaussian (1.70 Hz) and the Circle targets ($BF_{10}=0.222$), for the Point (1.88 Hz) and CCP targets ($BF_{10}=0.324$), and for the Gaussian and Bessel (1.78 Hz, $BF_{10}=0.324$) targets. Other pairwise comparisons showed inconclusive evidence regarding whether microsaccade rate differs between the targets ($BF_{10}$ ranged from 0.383 to 1.99).

Regarding microsaccade displacement (Fig. 6B), a Bayesian ANOVA also best supported a model with only the main effects of fixation target and target polarity ($BF_m=41.5$). Microsaccade displacement was larger for black-on-grey (0.269 deg) than for white-on-black targets (0.238 deg, $BF_{10}=1515$). Pairwise comparisons showed that, across target polarities, the data supported that microsaccade displacement was larger for the Gaussian (0.307 deg) and Circle (0.313 deg) targets than for the other three fixation targets (Point: 0.227 deg, CCP: 0.228 deg, Bessel: 0.195 deg; $BF_{10}>411$). Microsaccade displacement was the same for the Gaussian and Circle targets ($BF_{10}=0.241$) and the Point and CCP targets ($BF_{10}=0.221$). Other pairwise comparisons showed inconclusive evidence regarding whether microsaccade displacement differs between the targets ($BF_{10}$ ranged from 0.997 to 2.48).

**Fig. 7:**
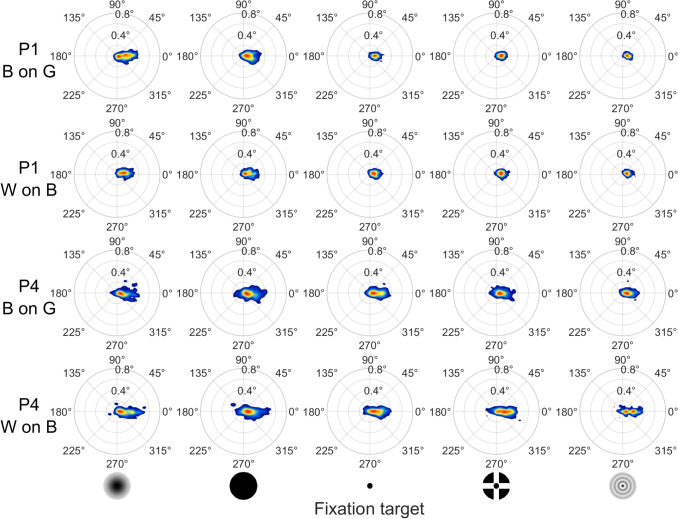
Histograms of drift displacement and direction. Histograms showing drift displacement and direction are plotted per fixation target and polarity condition for two example participants (P1 and P4). *B on G* refers to the black-on-grey polarity condition, and *W on B* to the white-on-black polarity condition

### Drift characteristics

We begin our examination of drift characteristics with the histograms in Fig. 7, which show the distribution of drift displacement and direction across target polarity and fixation targets for the same two example participants as in Fig. 5. The histograms show that drift was primarily rightward for both example participants. For all participants, drift had a single predominant direction, ranging from rightward to downward, with some exhibiting an oblique intermediate direction. Comparing the drift displacement in Fig. 7 (magnitude in the histograms) to the microsaccade displacement in Fig. 5, drift displacement appears to be about half that of the microsaccade displacement. This is consistent with the relative magnitudes of drift and microsaccade displacement reported by Bowers et al. (2021). Comparing drift and microsaccade directions, it can be seen that the predominant drift direction was opposite to one of the predominant microsaccade directions. This was observed for all participants. Neither drift displacement nor direction appears to differ systematically between targets and target polarities.

**Fig. 8:**
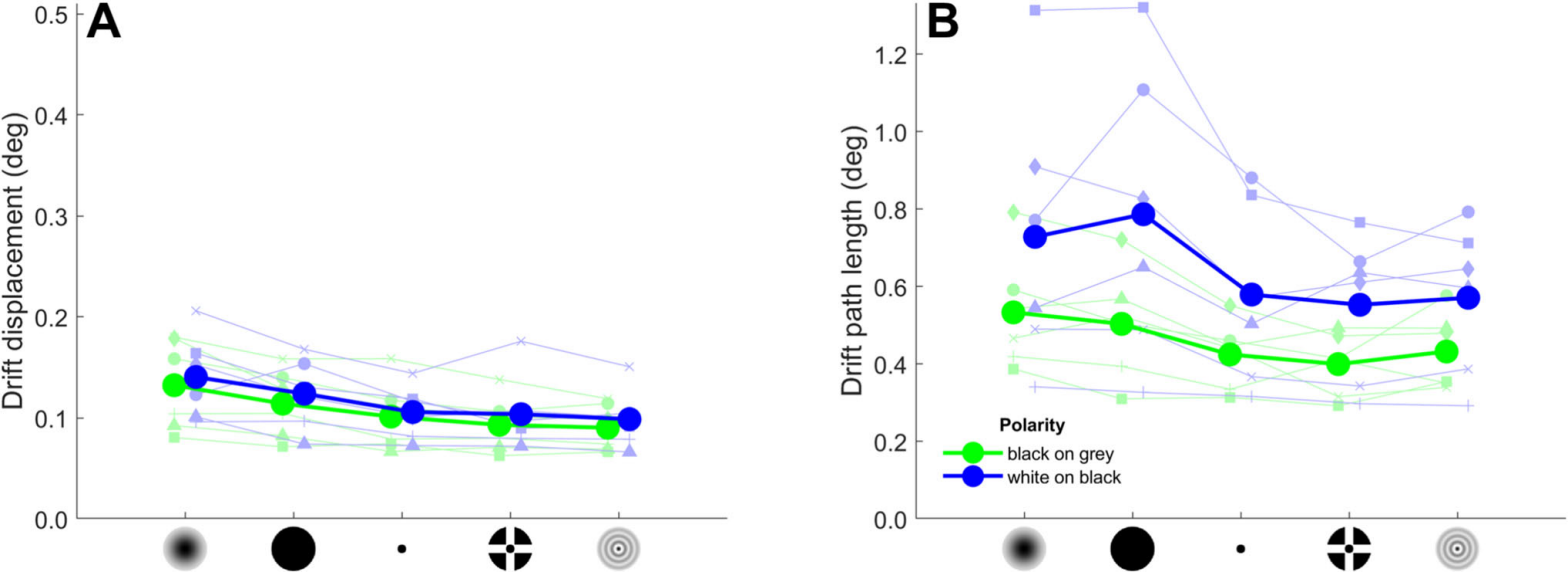
Fixational drift characteristics for the different fixation targets and polarity conditions. Shown are the drift displacement (**A**) and drift path length (**B**) for each fixation target and polarity condition, averaged across participants (*large filled circles connected by thick lines*). Also shown are the values for the six individual participants (*small faded symbols connected by thin lines*). The data for the two polarity conditions are horizontally offset for clarity

**Fig. 9:**
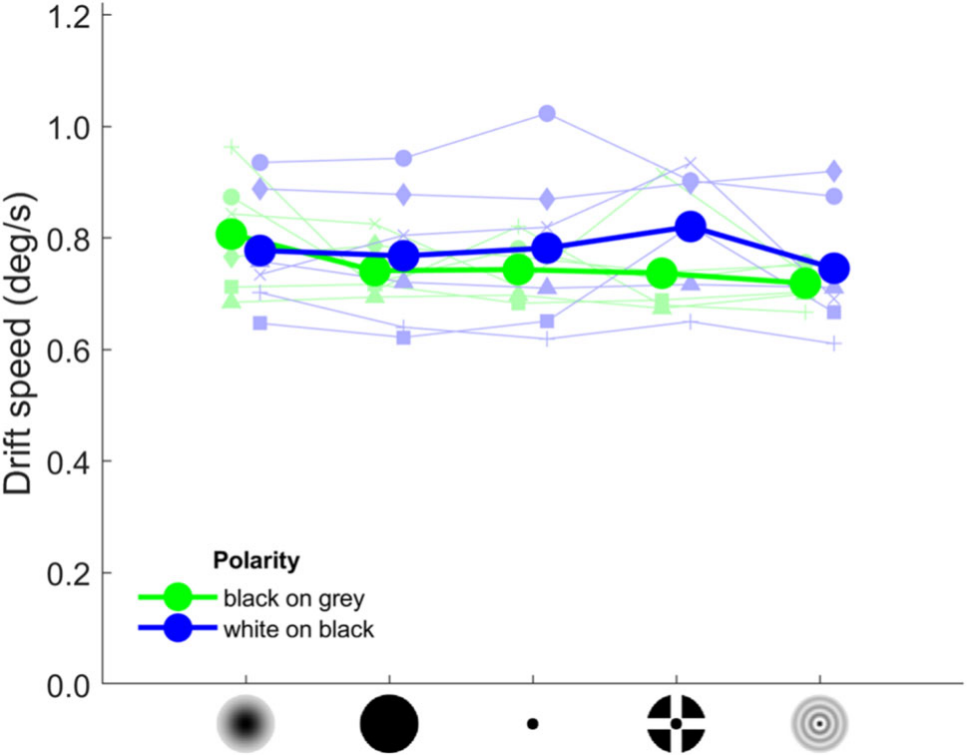
Fixational drift speed for the different fixation targets and polarity conditions, averaged across participants (*large filled circles connected by thick lines*). Also shown are the values for the six individual participants (*small faded symbols connected by thin lines*). The data for the two polarity conditions are horizontally offset for clarity

Figures 8 and 9 provide quantitative analyses of drift characteristics. A Bayesian ANOVA analysis of drift displacement (Fig. 8A) showed that a model with only the main effects of fixation target and target polarity was most supported by the data ($BF_m=4.13$), although only marginally more so than a model only including the main effect of fixation target ($BF_m=3.56$). Indeed, the data did not provide conclusive evidence that drift displacement is larger for black-on-grey (0.106 deg) than for white-on-black targets (0.115 deg, $BF_{10}=0.597$). Pairwise comparisons showed that, across target polarities, the data supported that drift displacement was larger for the Gaussian (0.137 deg) and Circle (0.119 deg) targets than for the other three fixation targets (Point: 0.104 deg, CCP: 0.0984 deg, Bessel: 0.0945 deg; $BF_{10}>20.26$, except inconclusive evidence for a difference between the Circle and Point targets, $BF_{10}=2.29$). Drift displacement was the same for the Point and CCP targets ($BF_{10}=0.322$) and the CCP and Bessel targets ($BF_{10}=0.244$). Other pairwise comparisons showed inconclusive evidence regarding whether drift displacement differs between the targets ($BF_{10}$ ranged from 0.586 to 0.831).

Drift path lengths (Fig. 8B) were much larger than the overall drift displacement, as expected since drift meanders along a complex path rather than take a straight line. A Bayesian ANOVA analysis of drift path length showed that a model with only the main effects of fixation target and target polarity was most supported by the data ($BF_m=33.5$). Drift path length was larger for white-on-black (0.644 deg) than black-on-grey targets (0.459 deg, $BF_{10}=0.597$). Pairwise comparisons showed that, across target polarities, the data supported that drift path length for the Gaussian (0.631 deg) and Circle (0.645 deg) targets was larger than for the CCP target (0.502 deg), while it did not provide evidence supporting that drift path length for these two targets was different than for the Point (0.476 deg) and Bessel (0.502 deg) targets. The drift path length was the same for the Gaussian and the Circle targets ($BF_{10}=0.225$), and for the CCP, Point and Bessel targets ($BF_{10}$ ranged from 0.220 to 0.279).

Regarding drift speed (Fig. 9), the null model was better supported by the data ($BF_m=6.70$) than models that included a main effect of fixation target ($BF_m=0.058$) or target polarity ($BF_m=2.17$). As such, the data provide evidence that drift speed does not differ between fixation targets or target polarities. It should be noted that, given the constant drift speed across fixation targets and target polarities, drift path length (Fig. 8B) is roughly a mirror image of the microsaccade rate (Fig. 6A): lower microsaccade rates, and thus longer intersaccadic intervals coincide with longer drift path lengths.

## Discussion

We used a high-precision retinal eye tracker to examine fixational eye movements during sustained fixation of five different fixation targets in two target polarity conditions. Our aim was to examine whether there were differences in overall fixational stability between these targets and target polarities and to further examine differences in the characteristics of the eye movements (microsaccades and drift) that occur during fixation. Examining the overall fixation stability by means of the spatial spread of gaze positions (operationalized as the BCEA) during 30-s trials, we found that fixation was more stable for targets with a small feature in the center (the Point, Circle-Cross-Point [CCP], and Bessel targets) than for targets that did not have this feature. We furthermore found that fixation was more stable for white-on-black than for black-on-grey fixation targets.

Examining the characteristics of microsaccades and drift during fixation, we found that there was no difference in drift speed or displacement during a drift episode between the target polarity conditions. Consistent with the findings for overall fixation stability, participants made more frequent and larger microsaccades when fixating black-on-grey targets than white-on-black targets. Further consistent with the overall fixation stability findings, we found that participants exhibited lower microsaccade displacement, drift displacement, and drift path length for targets with a small feature in the center. Drift speed, however, did not differ between targets, and microsaccade rate appeared to be slightly higher for the CCP target, and to some extent the Point target, than for the other fixation targets. Finally, we found that drift displacement was 2–3 times smaller than microsaccade displacement.

What is the best fixation target? In summary, by most measures we examined, the fixation targets that yield the most stable fixation are those with a small feature in the center (the Point, CCP, and Bessel fixation targets). These targets showed the lowest overall dispersion of gaze positions (BCEA) during the fixation episode and also consistently smaller microsaccade and drift displacement, and lower drift path length. However, if one wishes to minimize the microsaccade rate, then larger fixation targets without such a small feature in the center (the Gaussian and Circle targets) may offer a better choice. As such, we cannot conclude that there is a single ‘best’ fixation target. Instead, which is the most suitable target depends on whether one wishes to optimize for best overall fixation stability and lowest microsaccade and drift displacements, or for lowest microsaccade rate (e.g., to minimize electric or neural activity associated with saccades, Dimigen, Valsecchi, Sommer, & Kliegl, 2009). It is conceivable that for different study designs, one may opt to optimize for different fixational eye movement behaviors. Furthermore, regardless of which aspect of fixational eye movements one optimizes for, our results show that there are multiple fixation target designs that yield approximately the same behavior.

It should be noted that the differences between targets we report across the various measures are, in our opinion, neither surprising nor internally inconsistent. Given that the drift speed was constant across conditions, one would expect a larger drift path length and displacement for those target designs and polarities for which the microsaccade rate is lower, since lower microsaccade rates entail longer drift periods. This in turn may elicit larger average microsaccade displacement, as larger microsaccades may be required to counteract the larger drift displacement and stop gaze from wandering away from the fixation target. These larger drift and microsaccade displacements are also reflected in larger overall spread of the measured gaze positions (BCEA).

How do our findings relate to the literature? First, our conclusions only partially align with Thaler et al. (2013). They found that the CCP target yielded both a low microsaccade rate and low spatial spread during the fixation episode, which led them to label it as the ‘best’ fixation target. Like Thaler et al. (2013), we found that the spatial spread was lower for the CCP target than for the Circle target. However, unlike their findings, we observed higher – rather than lower – microsaccade rates for the CCP target than for the Circle target. The reason for this discrepancy is unclear, but it is worth noting that our findings do align with those of Steinman (1965) and McCamy et al. (2013), who reported more frequent microsaccades for smaller targets. It may be possible that, consistent with the report of McCamy et al. (2013) and the findings we report here, Thaler et al.’s participants made smaller microsaccades when fixating the CCP than the Circle target. The lower microsaccade rate they observed may have been caused by less reliable detection of these smaller microsaccades, given the limited precision of their eye-tracking equipment. Second, our finding that fixational stability was lower for black-on-grey than white-on-black fixation targets would not be expected from the literature, which showed no effect of contrast on fixational stability (Ukwade & Bedell, 1993; McCamy et al., 2013). It also runs counter to the expectation we expressed in the introduction that the edges of the grey background serve as an additional feature that could help fixational stability. While we have no explanation for this finding, it is possible that the more vivid after images of the target experienced in the white-on-black than the black-on-grey condition may aid keeping gaze aligned with the target.

Our finding that drift displacement was 2–3 times smaller than microsaccade displacement may appear surprising. One may expect the drift and microsaccade displacements to be similar or gaze would systematically wander away from the fixation target during a 30-s trial. Our finding aligns with the relative magnitudes of drift and microsaccade displacement reported by Bowers et al. (2021). What may explain these observations of a larger average displacement of microsaccades compared to drift? While the role of microsaccades is often presumed to be to counteract drift and bring gaze back to the target, participants do not only make microsaccades in the opposite direction as drift. For instance, participant P4 in Fig. 5 made frequent microsaccades in two directions, both in the same and the opposite direction as drift. In fact, four of the six participants in our study made frequent microsaccades in two opposing directions. We presume that the average displacement vector of all microsaccades would be in roughly the opposite direction as the average drift displacement, explaining why gaze did not significantly wander away from the target during fixation. Further examining the gaze position signals, we found that during fixation, participants who exhibited two predominant microsaccade directions also appeared to display square-wave jerks (SWJs, Abadi, & Gowen, 2004; Leigh, & Zee, 2006; Otero-Millan et al., 2011; McCamy et al., 2013), i.e., saccades away from the target followed at short latency by a second corrective saccade back to the target. Since the saccadic displacement during a SWJ has been reported to be larger than the displacement of saccades that are not part of a SWJ (Otero-Millan et al., 2011), it is possible that the presence of SWJs explains why we observe an average microsaccade displacement that is larger than the average drift displacement. Besides this potential explanation for the mismatch in drift and microsaccade displacement, our data does not allow us to rule out the possibility that the larger microsaccade amplitude was due to the microsaccades overshooting the center of the fixation target, as has been previously reported to occur (Tian et al., 2016, 2018; Willeke et al., 2019).

Even if our findings reveal that Thaler et al. ’s (2013) CCP fixation target is not unequivocally the ‘best’, do we recommend people to keep using it? Yes, since we think that our results do not indicate that the CCP would be an ineffective fixation target. What do we see as the advantages of the CCP fixation target? First, we like that it has a small detail at its center in terms of a clearly defined point. Such small points seem to elicit more stable fixation, and it is easy to instruct participants to fixate this point accurately. One potential enhancement to the CCP fixation target that may elicit even more stable fixation is to make the center point even smaller (cf. Hooge et al., 2025). It should be noted that it is not known whether further shrinking the center point of the CCP fixation target elicits more stable fixation, nor whether it might lead to a further and potentially undesirable increase in microsaccade rate. Second, another good aspect of the CCP fixation target is that it includes a large feature that makes the target easy to detect peripherally, which may be important for instance in paradigms where a fixation target jumps from one position to the next over considerable distances. Lastly, the inclusion of the cross in the fixation target design provides additional high-contrast edges that may help binocular fixation. A potentially negative aspect of the CCP fixation target is that its high-contrast edges can induce strong afterimages. If this is a concern, the Bessel fixation target with its smoothly modulated edges may be more suitable.

Another fixation target design dimension that could be explored is to use dynamic fixation points. For instance, some ophthalmologic devices, such as the Heidelberg Spectralis platform use a blinking dot as a fixation target, while retinal eye-tracking techniques using a visible scanning pattern that appears to the participant as a pattern of concentric inward-moving circles have also been reported (Damodaran et al., 2017; Vienola et al., 2018). It is unknown whether such dynamic fixation targets yield different fixational eye movements from the static targets we used in this study. To the best of our knowledge, only one study (Bowers et al., 2021) has examined fixational eye movements to a dynamic fixation target (concentric circles moving inward), but found no difference in fixational eye movements between this target and two different static targets. We think that this not only is an interesting design space to further explore, but also that such explorations are important given the use of dynamic fixation points in clinical practice.

Another aspect that one may wish to consider when assessing the quality of a fixation target is whether it elicits accurate fixation, that is, whether participants accurately look at its center. The methods of this study were not suitable for addressing this question. While the FET eye tracker we used is calibration-free, it does require the zero point to be determined for it to provide an accurate measure of gaze position. While each data collection started with four short fixations of a fixation target at the center of the display, we do not think this allows us to determine the zero accurately enough to address the accuracy of fixation to our fixation points. Other methods, such as gaze-contingent offset correction (O’Regan, 1978; Poletti & Rucci, 2016) would likely be required to determine the absolute fixation position with sufficient accuracy. This leaves fertile ground for future study.

Finally, we wish to highlight that we think it would not have been possible to conduct the current study with a video-based eye tracker. Many of the eye movements we report in this study are very small (e.g., mean drift and microsaccade displacements of 0.11 deg and 0.25 deg, respectively), and the differences between conditions are even smaller. For instance, the largest difference in drift amplitude between fixation targets (the Gaussian and Bessel targets) was only 2.55$^{\prime }$ (0.043 deg), and the average drift speed across fixation targets and target polarities was only 0.76 deg/s. To reliably measure such small and slow eye movements, an eye tracker with a precision much higher than that of even the best video-based eye tracker is required. Furthermore, the drift displacements we report are much smaller than the artefactual gaze shifts that are continuously induced in the gaze signal by, for instance, naturally occurring changes in pupil size (Hooge et al., 2025). As such, we join the call of other authors (e.g., Collewijn & Kowler, 2008; Poletti & Rucci, 2016) that researchers who plan to study miniature eye movements need to carefully consider whether the machinery they plan to use is suitable for their study design (cf. Nyström et al., 2025).

## Data Availability

The eye-tracking data and routines to make the result figures in the article are available from: https://github.com/dcnieho/NiehorsteretalBestFixationTarget.

## Declarations

### Open Practices Statement

The eye-tracking data and routines to make the result figures in the article are available from: https://github.com/dcnieho/NiehorsteretalBestFixationTarget. The experiment was not preregistered.
